# Oncological outcomes in minimally invasive vs. open distal pancreatectomy: a systematic review and network meta-analysis

**DOI:** 10.3389/fsurg.2024.1369169

**Published:** 2024-06-11

**Authors:** Nicky Zhun Hong Wong, Dominic Wei Ting Yap, Sherryl Lei Ng, Junie Yu Ning Ng, Juanita Jaslin James, Alfred Wei Chieh Kow

**Affiliations:** ^1^ Yong Loo Lin School of Medicine, National University of Singapore, Singapore, Singapore; ^2^ National University Centre for Organ Transplantation, National University Health System, Singapore, Singapore; ^3^ Division of Hepatobiliary and Pancreatic Surgery, Department of Surgery, National University Hospital Singapore, Singapore, Singapore

**Keywords:** pancreatectomy outcomes, pancreatic ductal adenocarcinoma (PDAC), open distal pancreatectomy, laparoscopic surgery, robotic surgery

## Abstract

**Background:**

Advancements in surgical techniques have improved outcomes in patients undergoing pancreatic surgery. To date there have been no meta-analyses comparing robotic and laparoscopic approaches for distal pancreatectomies (DP) in patients with pancreatic adenocarcinoma (PDAC). This systematic review and network meta-analysis aims to explore the oncological outcomes of laparoscopic distal pancreatectomy (LDP), robotic distal pancreatectomy (RDP) and open distal pancreatectomy (ODP).

**Methods:**

A systematic search was conducted for studies reporting laparoscopic, robotic or open surgery for DP. Frequentist network meta-analysis of oncological outcomes (overall survival, resection margins, tumor recurrence, examined lymph nodes, administration of adjuvant therapy) were performed.

**Results:**

Fifteen studies totalling 9,301 patients were included in the network meta-analysis. 1,946, 605 and 6,750 patients underwent LDP, RDP and ODP respectively. LDP (HR: 0.761, 95% CI: 0.642–0.901, *p* = 0.002) and RDP (HR: 0.757, 95% CI: 0.617–0.928, *p* = 0.008) were associated with overall survival (OS) benefit when compared to ODP. LDP (HR: 1.00, 95% CI: 0.793–1.27, *p* = 0.968) was not associated with OS benefit when compared to RDP. There were no significant differences between LDP, RDP and ODP for resection margins, tumor recurrence, examined lymph nodes and administration of adjuvant therapy.

**Conclusion:**

This study highlights the longer OS in both LDP and RDP when compared to ODP for patients with PDAC.

**Systematic Review Registration:**

https://www.crd.york.ac.uk/, PROSPERO (CRD42022336417).

## Introduction

The introduction of minimally invasive techniques has advanced the field of pancreatic surgery in recent decades (1–3). Despite the increase in procedures performed, LDP and RDP continue to present unique technical challenges for the surgeon (4–7).

Previously published meta-analyses have demonstrated that minimally invasive distal pancreatectomy (MIDP) is associated with lower morbidity and comparable oncological outcomes (overall survival, R0 resection, lymph node yield, use of adjuvant therapy) when compared to ODP (8–15). Whilst RDP appears to be comparable to LDP in terms of safety, to date no studies have compared oncological outcomes between laparoscopic distal pancreatectomy (LDP), robotic distal pancreatectomy (RDP) and open distal pancreatectomy (ODP) (16).

We performed a network meta-analysis on the studies reporting ODP, LDP and RDP in patients with histologically confirmed PDAC with the aim of clarifying if LDP or RDP improve oncological outcomes over ODP.

## Methods

**Figure 1:**
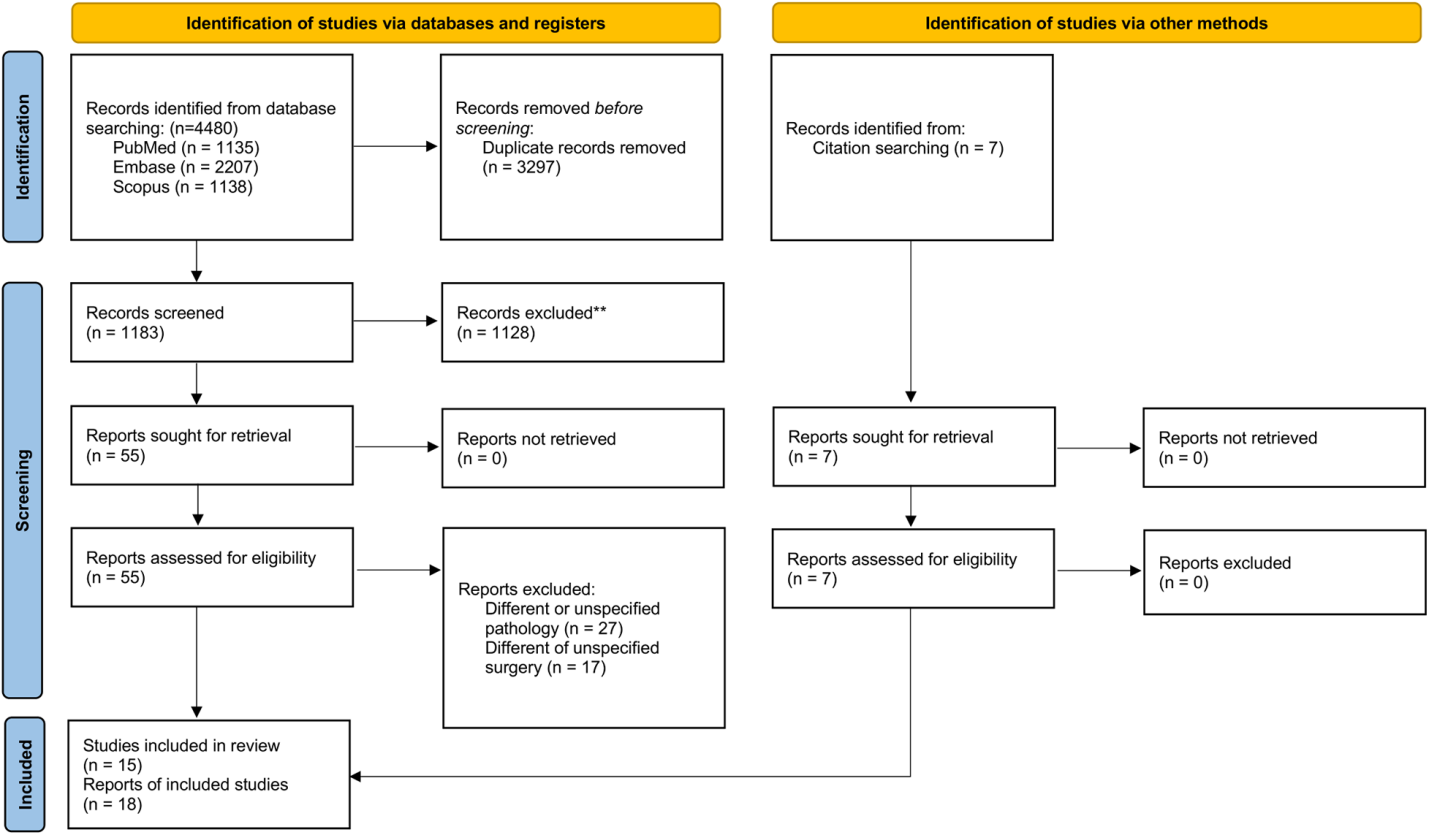
PRISMA flow diagram.

This review is registered on PROSPERO (CRD42022336417) and is reported in accordance with the 2020 Preferred Reporting Items for Systematic Reviews and Meta-Analysis (PRISMA) guideline. The PRISMA checklist is included in Figure 1 (17).

### Search strategy

A systematic search of PubMed, EMBASE and the Cochrane Library databases were conducted from inception til 7 July 2022 by two independent investigators (NWZH, SNL). The search terms used were “keyhole” or “robot” or “laparoscopic” or “minimally invasive” or “daVinci”, and “Pancreaticoduodenectomy” or “Whipple” or “pancreatectomy” or “pylorus-preserving pancreaticoduodenectomy” or “duodenopancreatectomy” or “jejunopancreatectomy” individually or in combination. Search terms used for this review are presented in Supplementary Table S1. A thorough manual search of reference lists in eligible studies was also performed.

### Eligibility

Key eligibility criteria included: (1) studies reporting the comparison of surgical techniques in human subjects receiving distal pancreatectomy and (2) studies reporting oncological outcomes (overall survival, positive resection margins, number of lymph nodes examined, tumor recurrence) and (3) studies that included pancreatic ductal adenocarcinoma. Exclusion criteria were: (1) Conference abstracts, reviews, case reports; (2) studies where type of MIDP was not specified; (3) studies that included other types of pancreatic surgeries.

### Study selection

Two reviewers (NZHW and SNL) independently screened and selected potentially eligible studies based on title and abstract. Full-text evaluation was independently performed by two reviewers (NZHW and SNL). Any conflicts between authors were discussed and resolved by a third independent reviewer (DWTY).

### Risk of bias assessment

As all included studies were observational, we used the Newcastle-Ottowa Scale (NOS) to evaluate the risk of bias. The studies were deemed to have high (<5 stars), moderate (5–7 stars) or low (≥8 stars) risk of bias (Supplementary Table S2).

### Statistical analysis

**Figure 2:**
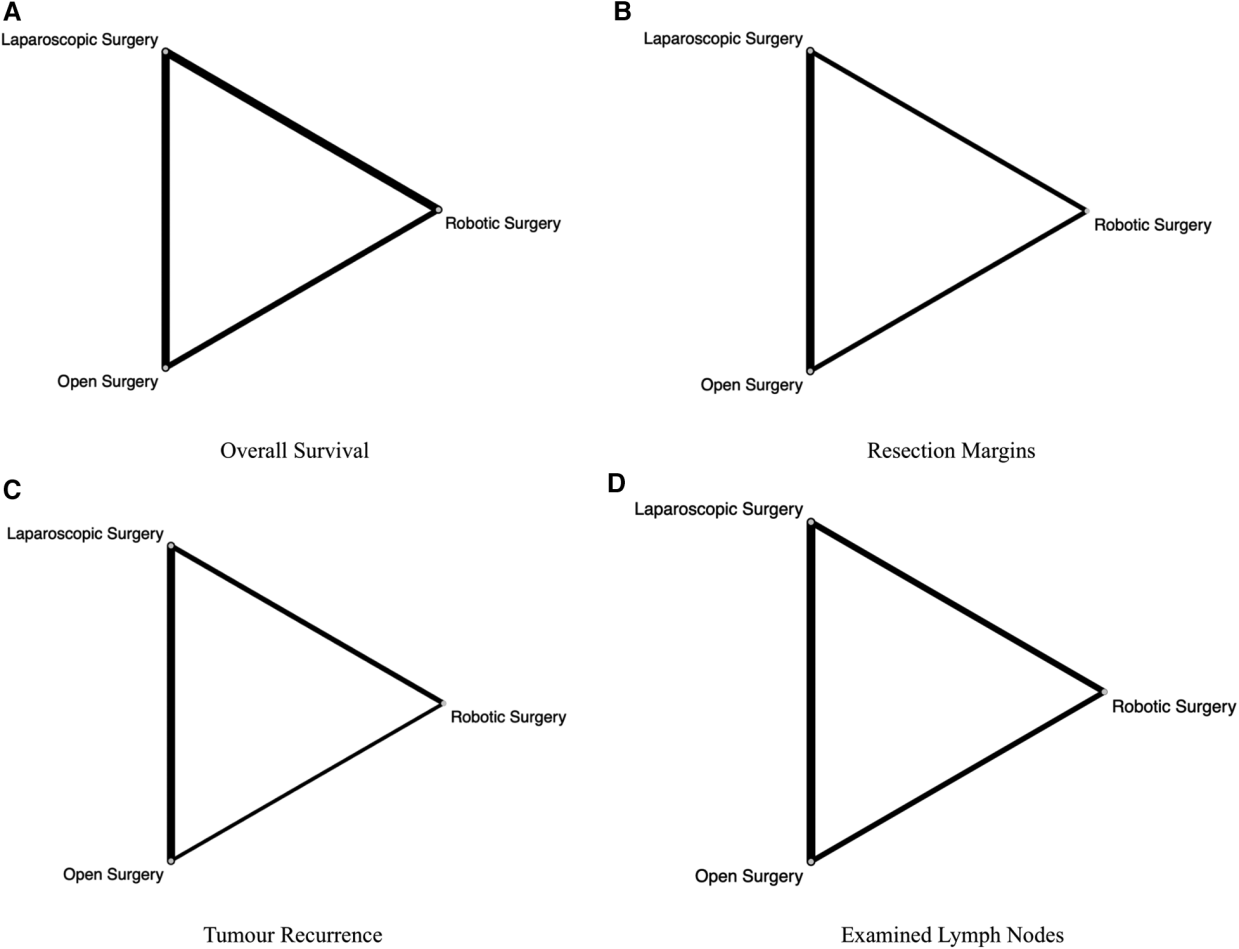
Network plot for comparisons amongst ODP, RDP and LDP for (**A**) overall survival, (**B**) resection margins, (**C**) tumor recurrence, (**D**) examined lymph nodes. The number of studies comparing connected surgical approaches is proportional to the width of the lines in the Network plot.

A frequentist network meta-analysis was employed to compare ODP, LDP and RDP. The network meta-analysis is a statistical approach that combines both direct and indirect evidence to allow for comparison between 3 or more interventions. Relative effects estimates between pairs within the network are more precise than single direct and indirect estimates (18). Treatments were ranked using the *P*-score provided by the *netmeta* package (19–21). A probability of ranking of 0.9 was considered high enough to be confidently reported as the correct ranking position of a surgical approach (22). Funnel plots of treatment estimates were visually inspected. Evidence of asymmetry or points lying outside 95% pseudo-confidence limits was interpreted as publication bias. Network plots of treatments (nodes) and comparisons (lines) were generated (Figure 2). Networks were examined for the inconsistency by the fitting of net splitting models (23). A *p* value of <0.05 was deemed to represent significant inconsistency between the direct and indirect estimate. A separate meta-analysis with meta-regression was performed by considering the proportion of patients with vascular resection when comparing positive resection margins between ODP and MIDP (RDP or LDP).

Hazard ratios (HR) and odds ratios (OR) were reported for categorical outcomes, whilst weighted mean differences (WMD) were reported for continuous data. Mean and standard deviation estimates were derived from studies that reported medians using methods described by Wan et al. and Luo et al. (24, 25). The random effects, restricted maximum likelihood (REML) method was used for the meta-analysis of outcomes. Results were deemed to be statistically significant if the 95% confidence interval did not cross the no-effect line (1 for binary outcomes and 0 for continuous outcomes). A *p* value of <0.05 was considered to be statistically significant. Data analysis was performed using R Statistical software (R 4.1.3).

### Outcomes

The primary outcome was overall survival. Secondary outcomes included positive resection margins, number of lymph nodes examined and tumor recurrence.

## Results

### Study selection

The electronic search returned 4,480 publications: 3,297 duplicates were excluded and 1,183 publications were screened. Of those, 1,128 were excluded after reviewing titles and abstracts and forty-four studies were excluded after reviewing full-text articles. An additional six studies were included from searching through reference lists. Nineteeen studies met the eligibility criteria. Two studies were further excluded due to overlapping inclusion periods in the National Cancer Database (NCDB) and one study was excluded due to double reporting of a study (26–28). Fifteen studies were included in the final analysis (Supplementary Table S2) (29–43).

### Study characteristics

All fifteen studies were retrospective observational studies (Table 1). One study compared all three approaches. Five studies utilised propensity score matching in their analysis (26, 30, 36, 42, 43). ODP and LDP were directly compared in ten studies. RDP and LDP were compared in three studies. ODP and RDP were compared in two studies. A summary of all comparisons made in the network meta-analysis are presented in Table 2.

**Table 1:** Summary of included studies.

Author	Year	Country	Design	Pathology	Patients (*n* = 9,301)	ODP	LDP	RDP	Age	ASA grade 1 (%)	BMI	Male sex (%)	Follow-up duration (years)	Neoadjuvant Therapy (%)	T3/T4 tumors (%)	Tumor size (cm)
Kooby	2010	United States	RCS	PDAC	212	189	23	—	LDP: 65.1 ± 12.3 ODP: 65.5 ± 11.3	nr	LDP: 28.5 ± 5.7 ODP: 26.2 ± 6.0	LDP: 12 (52.2) ODP: 80 (42.3)	6	nr	nr	LDP: 3.5 ± 1.3 ODP: 4.5 ± 2.8
Shin	2014	South Korea	RCS	PDAC	150	80	70	—	LDP: 61 (39–86) ODP: 65 (45–81)	LDP: 30 (42.9) ODP: 31 (38.8)	LDP: 24 (17–30) ODP: 23 (15–29)	LDP: 47 (67.1) ODP: 48 (60.0)	5	nr	LDP: 67 (95.7) ODP: 75 (93.8)	LDP: 3.0 (0.4–8.5) ODP: 3.5 (0.5–14.0)
Sulpice	2015	France	RCS	PDAC	2,753	2,406	347	—	LDP: 60.6 ± 14.7 ODP: 64.5 ± 12.0	nr	nr	LDP: 151 (43.5) ODP:1,170 (48.6)	5	nr	nr	nr
Zhang	2015	China	RCS	PDAC	51	34	17	—	LDP: 60 (44–75) ODP: 64 (40–76)	LDP: 9 (52.9) ODP: 15 (44.1)	LDP: 23 (18–28) ODP: 24 (19–29)	LDP: 11 (64.7) ODP: 19 (55.9)	6	nr	LDP: 14 (74.5) ODP: 30 (88.2)	LDP: 3.5 (2.3–5.5) ODP: 3.9 (1.8–5.5)
Stauffer	2016	United States	RCS	PDAC	72	28	44	—	LDP: 72 (55–90) ODP: 67 (44–85)	nr	LDP: 28 (17–63) ODP: 26 (17–43)	LDP: 26 (59.1) ODP: 16 (57.1)	5	nr	LDP: 32 (72.7) ODP: 22 (78.6)	LDP: 3.6 (0.5–7.5) ODP: 4.5 (0.2–15)
Zhang	2017	China	RCS	PDAC	98	76	22	—	LDP: 55 ± 13 ODP: 60 ± 9	nr	LDP: 23.9 ± 2.7 ODP: 23.7 ± 3.3	LDP: 9 (40.9) ODP: 30 (39.5)	4	nr	nr	LDP: 3.6 ± 1.3 ODP: 4.4 ± 1.4
Bauman	2018	United States	RCS	PDAC	79	46	33	—	LDP: 66 ± 2 ODP: 66 ± 2	nr	LDP: 26.2 ± 0.8 ODP: 27.8 ± 0.9	LDP: 17 (51.5) ODP: 18 (39.1)	6	ODP: 16 (34.8) LDP: 0 (0)	nr	LDP: 3.3 ± 0.3 ODP: 4.0 ± 0.4
Qu	2018	China	RCS	PDAC	70	—	35	35	RDP: 58.1 ± 11.1 LDP: 57.8 ± 11.4	RDP: 19 (54.3) LDP: 22 (62.9)	RDP: 24.7 ± 4.1 LDP: 24.2 ± 3.7	RDP: 22 (62.9) LDP: 22 (62.9)	3	RDP: 0 (0) LDP: 0 (0)	RDP: 5 (14.3) LDP: 8 (22.9)	RDP: 4.5 ± 1.8 LDP: 4.4 ± 2.2
Raoof	2018	United States	RCS	PDAC	1,947	1,342	605	—	nr	nr	nr	LDP: 322 (53.2) ODP: 623 (46.4)	3	LDP: 26 (4.3) ODP: 119 (8.9)	LDP: 199 (32.8) ODP: 405 (30.1)	nr
Raoof	2018	United States	RCS	PDAC	704	—	605	99	nr	nr	nr	RDP: 45 (45.5) LDP: 322 (53.2)	3	RDP: 9 (9.1) LDP: 26 (4.3)	RDP: 64 (64.6) LDP: 418 (69.1)	RDP: 3.5 (2.5–4.5) LDP: 3.7 (2.6–5.0)
Baimas-George	2020	United States	RCS	PDAC	75	—	42	33	RDP: 68 (40–85) LDP: 71 (50–88)	RDP: 1 (3.0) LDP: 0 (0)	RDP: 27 (19–40) LDP: 25 (17–69)	RDP: 16 (48.5) LDP: 25 (59.5)	nr	RDP: 4 (12.12) LDP: 3 (7.1)	RDP: 23 (69.7) LDP: 33 (78.6)	RDP: 3.5 (1.5–7.2) LDP: 4.5 (0.4–9.7)
Magistri	2020	Italy, United States	RCS	PDAC	54	36	—	18	nr	nr	nr	nr	5	nr	RDP: 8 (44.4) ODP: 16 (44.4)	RDP: 1.8 (0.2–4.7) ODP: 1.0 (0.1–10.0)
Nassour	2020	United States	RCS	PDAC	2,718	2,386	—	332	RDP: 67 ± 13 ODP: 66 ± 12	nr	nr	RDP: 145 (43.7) ODP: 1,112 (46.6)	5	0 (0)	nr	nr
Chen	2021	China	RCS	PDAC	172	86	86	—	LDP: 62.7 ± 8.7 ODP: 62.9 ± 8.8	LDP: 40 (46.5) ODP: 41 (47.7)	LDP: 22.5 ± 2.5 ODP: 22.3 ± 2.3	LDP: 54 (62.7) ODP: 54 (62.7)	5	nr	LDP: 39 (45.3) ODP: 37 (43.0)	LDP: 4.1 ± 1.5 ODP: 4.2 ± 1.4
Chopra	2021	United States	RCS	PDAC	146	41	17	88	nr	RDP: 1 (1.1) LDP: 0 (0) ODP: 0 (0)	nr	RDP: 42 (47.7) LDP: 7 (41.2) ODP: 25 (61.0)	10	ODP: 24 (58.5) LDP: 4 (23.5) RDP: 46 (52.3)	RDP: 22 (25.0) LDP: 5 (29.4) ODP: 14 (34.1)	nr

PDAC, pancreatic ductal adenocarcinoma; nr, not reported; RDP, robotic distal pancreatectomy; LDP, laparoscopic distal pancreatectomy; ODP, open distal pancreatectomy; ASA, American Society of Anesthesiologist score; BMI, body mass index.

**Table 2:** Summary of comparisons included in the network meta-analysis.

	Direct comparisons	Participants	Publication years	Study location		
				America	Europe	Asia
LDP vs. ODP	9	5,534	2010–2021	4	1	4
RDP vs. ODP	2	2,772	2020	2	1	0
RDP vs. LDP	3	849	2018–2020	2	0	1
RDP vs. LDP vs. ODP	1	146	2021	1	0	0

A total of 9,301 patients were included in this analysis. 1,946 patients underwent LDP, 605 underwent RDP and 6,750 underwent ODP. Baseline characteristics of patients are included in Table 1.

Two studies reported data on Radical Antegrade Modular Pancreatosplenectomy (RAMPS) (36, 37). In both studies, RAMPS were performed more frequently in patients undergoing laparoscopic surgery.

Three studies reported data on vascular resection (37, 41, 42). There was a statistically significant difference in the rates of vascular resection between the three arms in one study with Chopra et al. reporting higher rates of vascular resection in the ODP group (41).

### Overall survival

**Figure 3:**
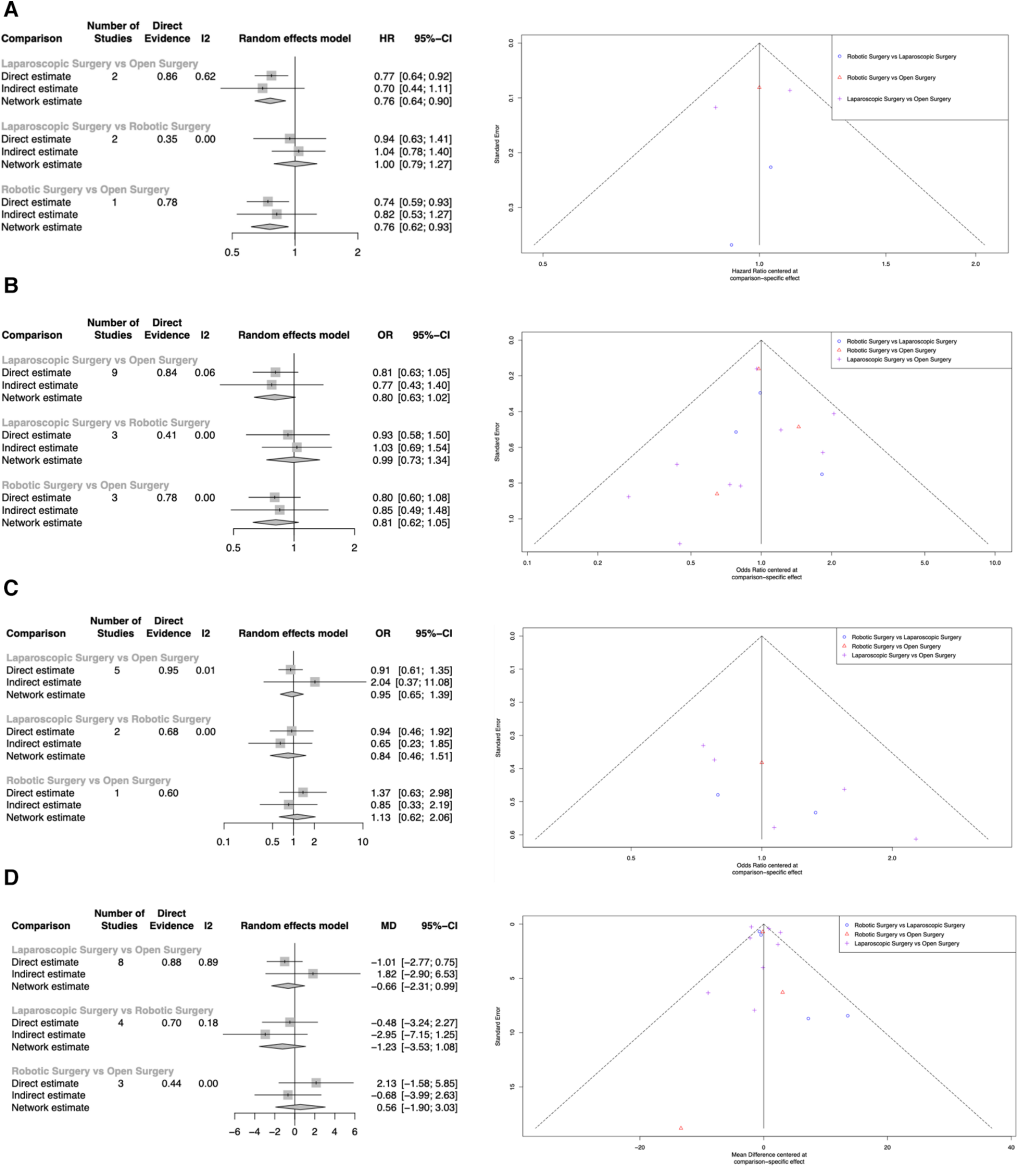
Network meta-analysis of LDP, RDP and ODP. (**A**) Overall Survival. (**B**) Resection Margins. (**C**) Tumor Recurrence. (**D**) Examined Lymph Nodes.

Overall survival was reported in five studies (29–32, 43). Both LDP (HR: 0.761, 95% CI: 0.642–0.901, *p* = 0.002) and RDP (HR: 0.757, 95% CI: 0.617–0.928, *p* = 0.008) were associated with better overall survival when compared to ODP. There was no statistically significant difference in overall survival between LDP and RDP (HR: 1.00, 95% CI: 0.793–1.27, *p* = 0.968) (Figure 3A: Overall survival). RDP and LDP were ranked first and second respectively for overall survival (Table 3). Two studies (29, 32) reported a follow-up duration of five years whilst three studies (30, 31, 43) reported a follow-up duration of three years. In this analysis, only three studies included information on tumor size of the PDAC. Qu et al. reported the median sizes of tumors to be 4.5 cm and 4.4 cm whilst Raoof et al. included tumours of 3.5 cm and 3.7 cm in the RDP and LDP groups respectively (31, 43). In another study by Raoof et al., the majority of tumors were ≥4 cm in both the ODP and LDP groups (30). All studies reported on tumour sizes found them to be comparable in the various arms of comparison.

**Table 3:** *P* ranking of treatments for outcomes of interest.

	1st	2nd	3rd
Overall survival	RDP, *p* = 0.756	LDP, *p* = 0.741	ODP, *p* = 0.002
Resection margins	LDP, *p* = 0.746	RDP, *p* = 0.709	ODP, *p* = 0.046
Tumour recurrence	LDP, *p* = 0.666	ODP, *p* = 0.525	RDP, *p* = 0.309
Examined lymph nodes	LDP, *p* = 0.818	ODP, *p* = 0.444	RDP, *p* = 0.238

### Resection margins

Resection margins were reported in thirteen studies (29–31, 33–42). Both LDP (OR = 0.803, 95% CI: 0.635–1.02, *p* = 0.068) and RDP (OR = 0.811, 95% CI: 0.623–1.05, *p* = 0.115) were not associated with higher rates of positive resection margins. There was no statistically significant difference in positive margins between LDP and RDP (OR =  0.990, 95% CI: 0.730–1.34, *p* = 0.950) (Figure 3B: Resection Margins). LDP and RDP were ranked first and second respectively for resection margins (Table 3). Positive margins were defined as tumor extension within 1 mm of the margin in 2 studies (34, 41), microscopic evidence of invasion in three studies (33, 39, 42) and microscopic or gross evidence of invasion in two studies (35, 37). Positive margins were not defined in the remaining six studies (29–31, 36, 38, 40).

In a separate analysis of three studies reporting both resection margins and vascular resections, MIDP (LDP or RDP) was not associated with a higher rate of positive resection margins (OR = 0.625, 95% CI: 0.078–5.03, *p* = 0.434). Meta regression considering proportion of patients with vascular resection did not reach a level of statistical significance (*p* = 0.425) (Supplementary Figure S1).

### Tumor recurrence

Tumor recurrences were reported in six studies. Both LDP (OR = 0.948, 95% CI: 0.647–1.39, *p* = 0.783) and RDP (OR = 1.13, 95% CI: 0.622–2.06, *p* = 0.684) were not associated with statistically significant higher rates of tumor recurrence. There was no statistically significant difference in tumor recurrence between LDP and RDP (OR = 0.838, 95% CI: 0.463–1.51, *p* = 0.561) (Figure 3C: Tumor recurrence). LDP and ODP were ranked first and second respectively for tumor recurrence (Table 3).

### Examined lymph nodes

The number of examined lymph nodes were reported in thirteen studies. Compared to ODP, LDP (WMD = −0.662, 95% CI: −2.31 to 0.989, *p* = 0.432) and RDP (WMD = 0.565, 95% CI = −1.90 to 3.03, *p* = 0.654) did not achieve a statistically significant difference in lymph node examined. Likewise, there was no statistically significant difference in lymph nodes examined between LDP and RDP (WMD = −1.23, 95% CI = −3.53 to 1.08, *p* = 0.300) (Figure 3D: Examined lymph nodes). LDP and ODP ranked first and second respectively for the number of examined lymph nodes (Table 3).

As some studies included skewed data as described by Shi et al, an additional sensitivity analysis was performed (44). Four studies with significantly skewed data were excluded. There were no statistically significant differences in examined lymph nodes between LDP (WMD = −0.591, 95% CI = −2.64 to 1.46, *p* = 0.572) and RDP (WMD = 0.526, 95% CI = −2.31 to 3.37, *p* = 0.719) when compared to ODP. Similarly, there was no statistically significant difference in lymph nodes examined between LDP and RDP (WMD = −1.12, 95% CI = −1.47 to 3.70, *p* = 0.397).

## Discussion and conclusion

Over the past decade, MIDP including the use of LDP and RDP has grown in popularity. With the increasing adoption of MIDP, the Yonsei criteria was described using several pathological factors to determine if minimally invasive approaches were suitable for tumors arising from the pancreatic body and tail (45). Studies from high volume centers have demonstrated that MIDP decreased the risk of complications compared to ODP (9). Despite this, the oncological benefits of LDP, RDP and ODP remain poorly understood. Our network meta-analysis compared oncological outcomes in fifteen cohort studies comparing ODP, LDP and RDP in over 9,000 patients with pancreatic adenocarcinoma. Both LDP and RDP demonstrated longer overall survival when compared to ODP. R0 resections, tumor recurrence and lymph nodes examined were comparable between all three interventions. While there may be potential selection bias in these retrospective studies, where tumor sizes may be different between different intervention groups (e.g., smaller tumours were offered MIDP as compared to ODP), we did not find any significant difference between the tumour size of the comparative groups in this analysis.

There have been limited studies with direct comparisons between RDP and other surgical approaches. However, through the indirect comparisons obtained in this network meta-analysis, we were able to show that oncological outcomes in RDP were comparable to those of ODP and LDP. Network meta-analyses combine direct evidence within studies and indirect evidence across studies to enable indirect comparisons of surgical techniques. The relative effectiveness of different surgical treatments may be assessed even if they have not been previously compared in individual RCTs. A network meta-analysis provides several benefits over a standard pairwise meta-analysis as treatment rankings with probabilities can be accessed. Results are more representative of the available evidence and are more reliable compared to pairwise meta-analysis (46, 47).

In a meta-analysis by Lyu et al., R0 margins were best achieved by RDP, robotic assisted distal pancreatectomy (RADP), LDP and ODP whilst lymph node harvest was best achieved by RDP followed by RADP, ODP and LDP (48). However the inclusion criteria differed between both studies. In our analysis only studies reporting PDAC were included in the analysis whilst the type of tumor was not defined by Lyu et al. Whilst Lyu et al.'s findings are generalizable to a greater degree of pathologies, our results are more pertinent to PDAC.

Given the lower rates of postoperative complications in MIDP and comparable oncological outcomes in RDP and LDP with ODP, MIDP should be recommended as the treatment of choice in experienced centres (48, 49). The LEOPARD RCT demonstrated that MIDP is associated with better functional recovery and post operative outcomes compared to ODP (50). It must be noted that the adoption and acceptance of minimally invasive techniques are also influenced by tumour characteristics, vascular involvement, logistical issues such as access to robots, and for institutions with lower case-load, prioritizing the education of younger residents in performing traditional open distal pancreatectomy over minimally invasive techniques (51, 52).

Results of this study should be interpreted with due consideration of some limitations. First, there are to date, no randomized controlled trials comparing oncological outcomes in all three surgical approaches for patients with pancreatic adenocarcinoma. Some studies mitigated this through the introduction of propensity matching, which has been shown to be able to adequately match patients to appropriate controls (53, 54). However, randomized controlled trials involving the three surgical approaches are still necessary for direct comparisons between interventions. Furthermore, heterogeneity exists in the majority of studies included in this network meta-analysis due to inherent differences in study populations, tumor factors and surgical experience (55). Although our results were limited to PDAC patients, outcomes continue to be influenced by molecular and metabolic subtypes within PDAC tumors, with basal and glycolytic subtypes demonstrating poorer prognosis (56–58). Lastly, our study was unable to account for other factors that are associated with OS such as nodal positivity, tumor stage, borderline resectable tumours, patient performance status, neoadjuvant and adjuvant therapy as well as pre and postresection tumor markers (59–63).

Resection margins in distal pancreatectomy encompass more than the neck of pancreas. Although R0 was most commonly defined as the absence of microscopic invasion at the surgical resection margins, most studies did not specify the exact definition used. Existing literature also revealed high variability in terms of rates of resections (64, 65). As a result we deemed R1 and R2 resections to be equivalent to positive margins to increase the generalizability of our results. However, the superior, inferior, anterior and posterior margins are all of importance. As pancreatic cancers in the body and tail often are infiltrative and present late, resectable lesions must be removed in a radical resection with clearance of as much surrounding tissues as possible, including the adrenals, parts of the colon or stomach if necessary. With highly skilled minimally invasive hepatobiliary surgical teams, these complex surgical approaches are achievable.

Our study demonstrated that both LDP and RDP was associated with longer OS when compared to ODP. Other oncological outcomes were comparable between all three groups. These results reflect the oncological safety of both minimally invasive approaches for PDAC and pave the way for both LDP and RDP to be recognized as the standard of care for PDAC in experienced centers.

## Data Availability

The original contributions presented in the study are included in the article/**Supplementary Material**, further inquiries can be directed to the corresponding author.
