# Supplementary material for: Oncological outcomes in minimally invasive vs. open distal pancreatectomy: a systematic review and network meta-analysis
Source: Front Surg. 2024 Jun 11;11:1369169. doi: 10.3389/fsurg.2024.1369169 (PMC11203400; doi:10.3389/fsurg.2024.1369169)

**Supplementary Materials**

Contents

Supplementary Table 1: Search terms

Supplementary Table 2: Summary of included studies

Supplementary Table 3: Net Splitting of direct and indirect evidence

Supplementary Figure 1: Bubble plot of Meta Regression analysis for positive resection margins

Supplementary Tables

Supplementary Table 1: Search Information

| Date of search | 7^th^ July 2022 |
| --- | --- |
| Databases | Pubmed, Embase, Scopus |
| Search string | (((Pancreatectomy[MeSH Terms] OR Whipple*[Title/Abstract] OR Pancreatico*[Title/Abstract] OR pancreatic resectio* [Title/Abstract] OR PPPD[Title/Abstract] OR Duodenopancreat*[Title/Abstract] OR Jejunopancreat*[Title/Abstract])) AND (keyhole*[Title/Abstract] OR robot*[Title/Abstract] OR laparoscop*[Title/Abstract] OR daVinci[Title/Abstract] OR 'minimally invasive' [Title/Abstract])) AND (match* OR compar* OR propensit* OR random*) |

Supplementary Table 2: Summary of Included Studies

| Author, Year | Country | Treatment | Population | Age | ASA grade 1 (%) | BMI | Male sex (%) | Follow-up duration (years) | Vascular Resection (%) | RAMPS | NOS |
| --- | --- | --- | --- | --- | --- | --- | --- | --- | --- | --- | --- |
| Kooby, 2010 | United States | LDP vs ODP | LDP: 23  ODP: 189 | LDP: 65.1 ± 12.3  ODP: 65.5 ± 11.3 | nr | LDP: 28.5 ± 5.7  ODP: 26.2 ± 6.0 | LDP: 12 (52)  ODP: 80 (42) | 6 | nr | nr | 7 |
| Shin, 2014 | South Korea | LDP vs ODP | LDP: 70  ODP: 80 | LDP: 61 (39-86)  ODP: 65 (45-81) | LDP: 30 (43)  ODP: 31 (39) | LDP: 24 (17-30)  ODP: 23 (15-29) | LDP: 47 (67)  ODP: 48 (60) | 5 | nr | LDP: 52 (74)  ODP: 38 (48) | 8 |
| Sulpice. 2015 | France | LDP vs ODP | LDP: 347  ODP: 2406 | LDP: 60.6 ± 14.7  ODP: 64.5 ± 12.0 | nr | nr | LDP: 151 (43)  ODP: 1170 (49) | 5 | nr | nr | 8 |
| Zhang, 2015 | China | LDP vs ODP | LDP: 17  ODP: 34 | LDP: 60 (44-75)  ODP: 64 (40-76) | LDP: 9 (53)  ODP: 15 (44) | LDP: 23 (18-28)  ODP: 24 (19-29) | LDP: 11 (65)  ODP: 19 (56) | 6 | nr | nr | 7 |
| Stauffer, 2016 | United States | LDP vs ODP | LDP: 44  ODP: 28 | LDP: 72 (55-90)  ODP: 67 (44-85) | nr | LDP: 28 (17-63)  ODP: 26 (17-43) | LDP: 26 (59)  ODP: 16 (57) | 5 | LDP: 3 (7)  ODP: 2 (7) | LDP: 12 (27)  ODP: nr | 8 |
| Zhang, 2017 | China | LDP vs ODP | LDP: 22  ODP: 76 | LDP: 55 ± 13  ODP: 60 ± 9 | nr | LDP: 23.9 ± 2.7  ODP: 23.7 ± 3.3 | LDP: 9 (41)  ODP: 30 (39) | 4 | nr | nr | 6 |
| Bauman, 2018 | United States | LDP vs ODP | LDP: 33  ODP: 46 | LDP: 66 ± 2  ODP: 66 ± 2 | nr | LDP: 26.2 ± 0.8  ODP: 27.8 ± 0.9 | LDP: 17 (52)  ODP: 18 (39) | 6 | nr | nr | 7 |
| Yu, 2018 | China | RDP vs LDP | RDP: 35  LDP: 35 | RDP: 59.9 ± 9.8  LDP: 57.8 ± 12.1 | RDP: 50 (58)  LDP: 20 (53) | RDP: 24.7 ± 4.1  LDP: 24.2 ± 3.7 | RDP: 40 (47)  LDP: 25 (66) | 3 | nr | nr | 7 |
| Raoof, 2018 | United States | LDP vs ODP | LDP: 605  ODP: 1342 | nr | nr | nr | LDP: 322 (53)  ODP: 623 (46) | 3 | nr | nr | 7 |
| Raoof, 2018 (2) | United States | RDP vs LDP | RDP: 99  LDP: 605 | nr | nr | nr | LDP: 322 (53)  RDP: 45 (45) | 3 | nr | nr | 7 |
| Baimas-George, 2020 | United States | RDP vs LDP | RDP: 33  LDP: 42 | RDP: 68 (40-85)  LDP: 71 (50-88) | RDP: 1 (3)  LDP: 0 (0) | RDP: 27 (19-40)  LDP: 25 (17-69) | RDP: 16 (48)  LDP: 25 (60) | nr | nr | nr | 6 |
| Magistri, 2020 | Italy, United States | RDP vs ODP | RDP: 18  ODP: 36 | RDP: 63 (19-81)  ODP: 63 (18-85) | nr | RDP: 25 (17-38)  ODP: 25 (17-38) | RDP: 33 (40)  ODP: 66 (40) | 5 | RDP: 1 (1)  ODP: 10 (6) | nr | 6 |
| Nassour, 2020 | United States | RDP vs ODP | RDP: 332  ODP: 2386 | RDP: 67 ± 13  ODP: 66 ± 12 | nr | nr | RDP: 145 (44)  ODP: 1112 (47) | 5 | nr | nr | 7 |
| Chen, 2021 | China | LDP vs ODP | LDP: 86  ODP: 86 | LDP: 62.7 ± 8.7  ODP: 62.9 ± 8.8 | LDP: 40 (47)  ODP: 41 (47) | LDP: 22.5 ± 2.5  ODP: 22.3 ± 2.3 | LDP: 54 (63)  ODP: 54 (63) | 5 | nr | nr | 8 |
| Chopra, 2021 | United States | RDP vs LDP vs ODP | RDP: 88  LDP: 17  ODP: 41 | nr | RDP: 1 (1)  LDP: 0 (0)  ODP: 0 (0) | nr | RDP: 42 (48)  LDP: 7 (41)  ODP: 25 (61) | 10 | RDP: 6 (7)  LDP: 0 (0)  ODP: 10 (24) | nr | 6 |

Abbreviations: RDP, Robotic Distal Pancreatectomy; LDP, Laparoscopic Distal Pancreatectomy; ODP, Open Distal Pancreatectomy; NOS, Newcastle-Ottawa Scale; nr, Not Reported; ASA, American Society of Anesthesiologist score; BMI, body mass index.

Supplementary Table 3: Net splitting of direct and indirect evidence

|  | LDP vs ODP | RDP vs ODP | RDP vs LDP |
| --- | --- | --- | --- |
| Overall Survival | p = 0.698 | p = 0.698 | p = 0.698 |
| Positive Margins | p = 0.894 | p = 0.858 | p = 0.746 |
| Tumour Recurrence | p = 0.363 | p = 0.445 | p = 0.560 |
| Examined Lymph Nodes | p = 0.271 | p = 0.267 | p = 0.336 |

Abbreviations: RDP, Robotic Distal Pancreatectomy; LDP, Laparoscopic Distal Pancreatectomy; ODP, Open Distal Pancreatectomy.

Supplementary Figure 1 Bubble plot of Meta Regression analysis for positive resection margins


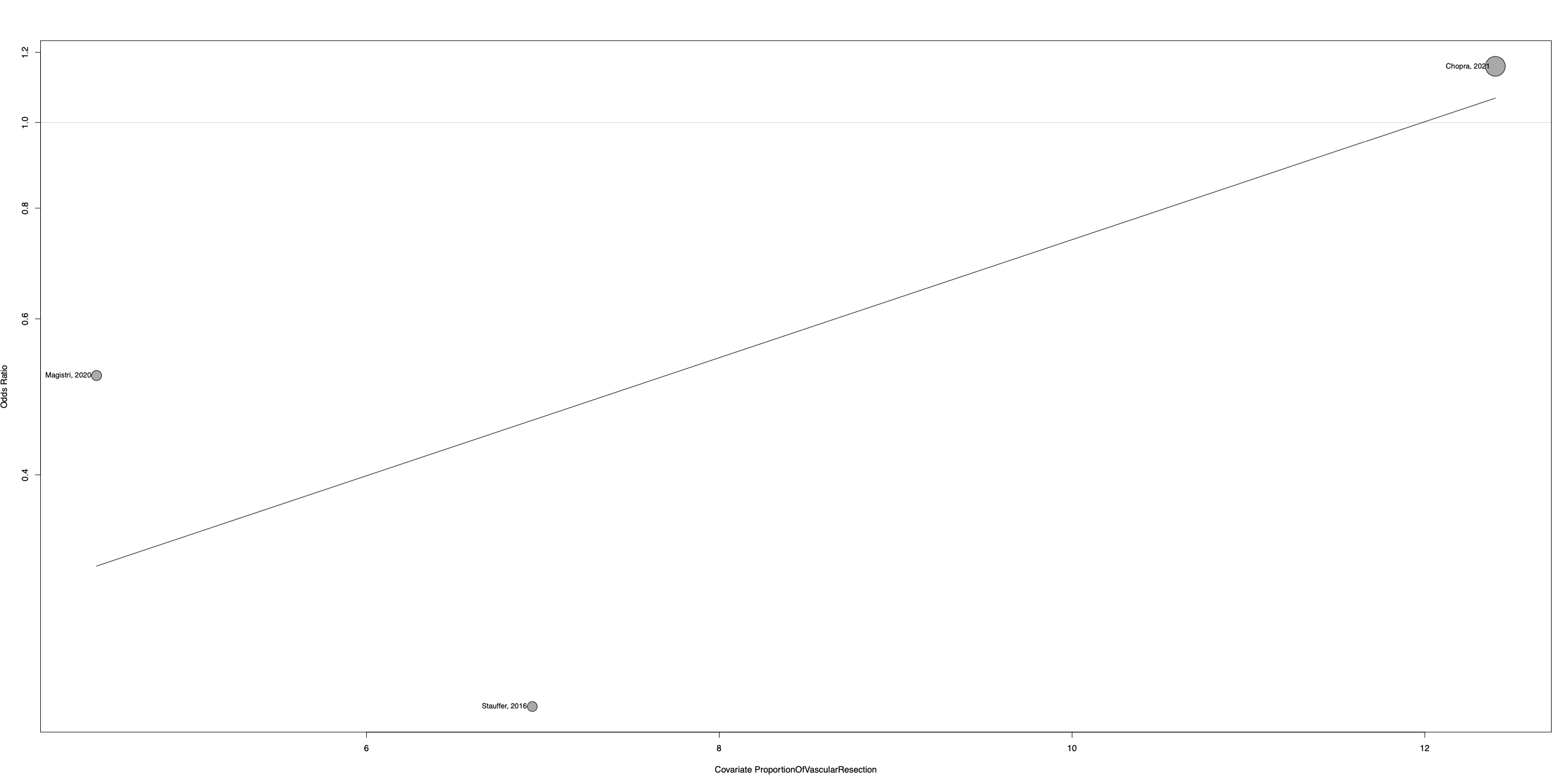

Supplement: Supplementary file 1 [file Datasheet1.docx]
